# Electrophysiology Study During Transcatheter Aortic Valve Replacement to Predict High-Degree Atrioventricular Block: An Unfinished Tale

**DOI:** 10.1016/j.shj.2024.100332

**Published:** 2024-06-25

**Authors:** Anne-Sophie Lacharite-Roberge, Kurt S. Hoffmayer

**Affiliations:** Division of Cardiology, Department of Medicine, Section of Cardiac Electrophysiology, University of California San Diego, La Jolla, California, USA

Percutaneous management of valvular heart disease, specifically transcatheter aortic valve replacement (TAVR), has evolved as a safe and efficacious option for the treatment of aortic stenosis.^1^ Although initially reserved for high-risk groups, TAVR is now performed in a younger and healthier population.^2^^,^^3^ Atrioventricular (AV) block is a known complication of TAVR, and a wide variety of management practices exist, including pre-emptive pacemaker implantation, observation, long-term rhythm monitoring, and in rare cases, electrophysiological testing. In 2020, the American College of Cardiology published a consensus decision pathway on conduction disturbances in patients undergoing TAVR. However, there remains paucity of adequately powered, randomized controlled trials on this topic.^4^ Recent data revealed an association between pacemaker implantation and all-cause death and heart failure rehospitalizations at 1 ​year following TAVR.^5^^,^^6^ This brings up the importance of evaluating pacing dependency and timing of the recovery of the AV node. Given the consideration of younger patients for TAVR, the implications of permanent pacing should undoubtedly be part of the preprocedural discussion and consent.

Electrophysiology (EP) testing has shown the His-ventricular (HV) as an invaluable measurement in predicting high grade AV block in patients with existing conduction abnormalities including left bundle branch block and bi-fascicular block.^7^

In this current issue of *Structural Heart: The Journal of the Heart Team*, Raad et al. describe the relevance of the post-TAVR HV interval in risk stratifying patients following TAVR. This was a single-center prospective study, and 121 patients underwent an EP study immediately before and after TAVR. Their findings revealed that a baseline right bundle branch block, a new persistent left bundle branch block, valve implant depth >4 mm, and a post-TAVR HV ≥65 ​ms were associated with a higher risk of high-degree AV block. In patients with pre-TAVR right bundle branch block or post-TAVR persistent left bundle branch block, no patients with post-TAVR HV <65 ​ms developed high-degree AV block. The authors analyzed a separate retrospective cohort to determine the time interval when the risk of high-degree AV block decreased to less than 5% in order to improve safe discharge parameters.

Only a limited number of studies have looked at invasive HV interval measurement, and there is no consensus on HV cutoff as a predictor of high-degree AV block post-TAVR.^8^ As the authors point out, this study is the largest cohort of patients undergoing TAVR and concomitant HV interval evaluation. However, the measurement of the HV interval immediately after deployment of the valve raises key questions. It is a well-known and experienced phenomenon in the EP community that in patients with and without existing bundle branch pathology, inadvertent catheter-induced trauma to the healthy bundle can lead to transient high-degree AV block.^9^ This is usually followed by progressive improvement and often recovery in His-Purkinje conduction, which may take several hours to days. Hence, it is dissatisfying that only a small number of patients underwent EP study 24 to 48 ​hours after TAVR. This may have yielded different and more favorable results from a conduction recovery standpoint, given a decrease in inflammation in the His bundle. Additionally, the authors note that patients who developed persistent high-degree AV block immediately following valve deployment were excluded from the analysis as they did not undergo a post-TAVR EP study. Although the decision for permanent pacing would have been the same, this information would have been useful to determine the type of block (nodal vs. infra-Hisian). Finally, the recommendation made by the authors for HV measurement immediately after TAVR may not be feasible at smaller community-based centers, and an erroneous measurement could have serious consequences if it influenced the decision for device implantation.

With the addition of an invasive HV measurement, we now possess several tools to predict the likelihood of high-degree AV block in patients undergoing TAVR. However, discussing long-term recovery of the conduction system and chronic pacing dependency is of utmost importance. Implantation of a pacemaker following TAVR is considered a routine procedure, and the decision to proceed with permanent pacing is often made liberally. Nonetheless, implications of long-term device placement, especially in a younger and lower-risk TAVR population, can have life-altering implications. Issues such as multiple generator changes, increased risk of infection, lead-associated complications, pacing-induced cardiomyopathy, and the possible need for extraction cannot be overlooked.^10^ Recovery of conduction has been consistently demonstrated in >50% of patients who underwent pacemaker implantation following TAVR.^11^^,^^12^ In 2012, De Carlo et al.^13^ published a study in the *American Heart Journal* regarding safety of a conservative strategy of permanent pacemaker implantation after TAVR. When safe and with guidance of EP, the authors opted to delay permanent pacemaker implantation despite the presence of a high-degree AV block following implantation. One-year survival was similar between patients who received permanent pacing and patients who did not, regardless of their preprocedural predictors of AV block. Although the authors of the TAVR-Conduction Study describe a novel way to predict acute high-degree AV block, it fails to discuss the decision-making process regarding permanent pacemaker implantation in these patients and the importance of short- and long-term monitoring given the high likelihood of conduction recovery. The authors note that 61 patients did wear an event monitor following TAVR implantation, but there is no data reported on monitor findings, long-term correlation with the HV interval, or device interrogation details in patients who did receive permanent pacing.

The authors report that 12 (10%) of patients developed high-degree AV block. Of those, 7 occurred immediately after valve deployment, 4 in the delayed postoperative period, and 1 after discharge. Several questions come to mind, in addition to future directions, when planning for management of acute AV block in a younger TAVR population. Did any patient recover conduction prior to discharge? Was temporary pacing used and the need for it assessed thoroughly before pacemaker implantation? Temporary transvenous pacemakers are regularly used for patients undergoing TAVR; however, one strategy that is rarely employed is the use of temporary permanent pacemaker. A recent small study published in 2023 titled “Feasibility study of temporary permanent pacemaker in patients with conduction block after TAVR” revealed that this is likely a safe approach to reduce premature permanent pacemaker implantation.^14^ In cases where permanent pacing is inevitable, pacemaker programming to minimize ventricular pacing is key to minimize the risk of pacing-induced cardiomyopathy.^11^ Finally, leadless pacing should be considered to avoid future issues with lead failure, infection, and generator changes in the correct population, given the high likelihood of conduction recovery.

In this current issue of *Structural Heart: The Journal of the Heart Team,* the TAVR-Conduction Study group reports on invasive HV measurement pre- and post-TAVR to predict high-degree AV block. AV block is a common complication of TAVR, and the measurement of the HV interval is a useful adjunct tool to risk stratify patients in the acute setting. Nonetheless, it remains unclear if the HV interval measured immediately after valve deployment is predictive of long-term pacing requirement. Further details regarding follow-up and monitoring of these patients are needed to make decisions regarding device implantation. Additionally, the feasibility of accurate HV measurement in the catheterization laboratory would be a challenge, especially in smaller centers. In the younger TAVR population, the decision to implant a permanent device has serious implications, and all cardiologists should work together to minimize implantation of unnecessary permanent devices.

## Funding

The authors have no funding to report.

## Disclosure Statement

The authors report no conflict of interest.
